# Wavelet Interactive Mamba Fusion Network for UAV Small Object Detection with Visible–Thermal Data

**DOI:** 10.3390/s26206625

**Published:** 2026-10-19

**Authors:** Qi Gao, Tianwen Guo

**Affiliations:** College of Computer and Information Engineering (College of Artificial Intelligence), Nanjing Tech University, Nanjing 211816, China; 202321047011@njtech.edu.cn

**Keywords:** Mamba, object detection, RGB-T, small objects, edge deployment

## Abstract

Accurate small object detection in low-altitude unmanned aerial vehicle (UAV) visible–thermal (RGB-T) scenarios remains challenging under complex illumination and background clutter due to thermal noise, weak cross-modal interaction, and attenuation of ultra-small target features. This paper proposes Wavelet Interactive Mamba Fusion Network (WIM-Net), a lightweight RGB-T detector with 10.4 million parameters. The network adopts a dual-branch backbone and introduces two modules for frequency-domain denoising and adaptive feature fusion. The Wavelet Interactive Mamba (WIM) module decomposes visible and thermal feature maps into multi-frequency sub-bands through a discrete wavelet transform and uses bidirectional Mamba interaction to model cross-modal dependencies with linear complexity. Instead of independent single-modal denoising, WIM uses low-frequency semantic cues to constrain high-frequency filtering, suppressing thermal noise while preserving target structures. The Cross-Modal Synergistic Fusion (CMSF) module builds bimodal mutual attention to adaptively combine visible texture and thermal radiation cues, reducing feature attenuation for tiny objects. Ablation studies show that WIM and CMSF contribute to noise suppression and small-object feature retention, respectively. Experiments on the RGBT-Tiny dataset demonstrate that WIM-Net achieves the best mean average precision at an intersection-over-union threshold of 0.5 (mAP50) of 52.0% and Precision of 79.9% among the evaluated single-modal and RGB-T detectors with only 10.4 million parameters, whereas its Recall and F1-score remain below those of QFDet and CALNet, reflecting a precision-oriented operating point. The compact architecture also preserves a favorable balance between detection accuracy and inference efficiency, with measured offline throughput on an NVIDIA Jetson Orin NX, while live airborne performance remains to be tested.

## 1. Introduction

In computer vision, RGB-T perception combines RGB images with thermal infrared maps to improve visual understanding under complex illumination. RGB images provide rich color and texture details, whereas thermal infrared maps capture object radiation cues, making RGB-T data useful for low-altitude remote sensing, autonomous driving perception, and night surveillance [1,2]. However, thermal infrared data acquired by practical sensors are often affected by noise, boundary blurring, and low spatial resolution due to object surface temperature variation, atmospheric interference, and sensor limitations. These degradations motivate the denoising and adaptive fusion design considered in this work.

Deep learning has improved both object detection and RGB-T feature representation, but the combination of thermal noise, modal imbalance, and tiny target scale remains difficult in low-altitude UAV scenes. Section 2 reviews the relevant detection and fusion methods in detail.

Despite this progress, low-altitude UAV RGB-T small object detection still faces several practical difficulties. Thermal infrared noise and boundary blurring can contaminate features during fusion, while insufficient adaptive interaction between visible texture and thermal radiation cues limits the exploitation of modal complementarity. Meanwhile, tiny objects occupying fewer than 16×16 pixels usually have weak responses and high similarity to background regions, leading to missed detections and false alarms [1,3,4]. In addition, many multimodal detection models contain a large number of parameters or require high computational cost, which makes real-time deployment on airborne edge devices difficult.

Motivated by these observations, this paper develops a lightweight RGB-T small object detection framework for low-altitude UAV scenarios. Instead of relying on direct feature concatenation or computationally heavy cross-modal interaction, the framework jointly considers frequency-domain representation, long-range dependency modeling, and adaptive complementary feature enhancement. The overall design aims to improve the robustness of visible–thermal feature representation while preserving tiny-object cues and maintaining deployment efficiency on airborne edge platforms.

The main contributions of this paper are summarized as follows:

- The WIM module turns cross-modal low-frequency semantics into soft gates that directly filter the high-frequency sub-bands before reconstruction and fusion, and this purify-first step is cascaded with the spatial-domain bidirectional mutual-attention fusion (CMSF) in a single compact detector of only 10.4M parameters, suppressing feature contamination caused by thermal infrared noise with linear complexity.
- The CMSF module uses bimodal bidirectional mutual attention for adaptive complementary enhancement of heterogeneous features and improves mAP50, although the full model has lower Recall than the CMSF-only variant.
- A lightweight RGB-T small object detector, WIM-Net, is evaluated on RGBT-Tiny and DroneVehicle, and its offline processing speed is measured on an NVIDIA Jetson Orin NX. Live airborne performance remains to be tested.

## 2. Related Work

### 2.1. Research Status of Object Detection

Object detection has progressed from handcrafted feature methods to CNN-based frameworks and, more recently, state-space model architectures.

Early detection algorithms relied on handcrafted features and acceleration techniques to cope with limited computing resources. Viola and Jones proposed the Viola–Jones detector [5], which integrated integral images, feature selection, and cascade classifiers to improve detection speed. Dalal and Triggs proposed the HOG detector [6], which became a representative feature-based object detector.

With the resurgence of CNNs, two-stage detectors were further developed and achieved higher detection accuracy. Girshick et al. proposed Regions with CNN features (R-CNN) for object detection [7], increasing the mAP on VOC07 from 33.7% to 58.5%. To reduce the slow inference caused by redundant feature computation, He et al. proposed the Spatial Pyramid Pooling Network (SPPNet) [8], which improved detection speed. Girshick proposed the Fast R-CNN detector [9], integrating the advantages of R-CNN and SPPNet to further improve speed. Ren et al. introduced the Region Proposal Network (RPN) and proposed Faster R-CNN [10], enabling end-to-end, near-real-time detection.

For higher inference efficiency, CNN-based one-stage detectors complete detection in a single step. Redmon et al. proposed YOLO in 2016 [11], improving detection accuracy while maintaining high detection speed. Liu et al. introduced multi-reference and multi-resolution detection techniques and proposed SSD [12], improving detection accuracy for small objects. Lin et al. proposed RetinaNet with Focal Loss [13], achieving accuracy comparable to that of two-stage detectors while maintaining high detection speed.

In recent years, the demand for efficient high-resolution image processing has drawn attention to the Mamba architecture. Gu and Dao proposed the selective state-space architecture Mamba [14], which efficiently processes long sequences with linear computational complexity. However, the native Mamba architecture is less tailored to local visual feature modeling and cross-modal interaction. Researchers have therefore proposed several Mamba-based variants. Wang et al. proposed Mamba YOLO with an ODMamba backbone for efficient object detection and a dedicated block for modeling local image dependencies [15]. For small object detection, Wu et al. combined Mamba-based global modeling, multi-scale feature enhancement, and context-guided upsampling in MV-YOLO [16]. Jiang et al. proposed a hybrid CNN-Mamba architecture to balance local fine-grained feature extraction and global long-range dependency modeling [17], while UAV-Mamba further optimized the state-space paradigm for aerial imagery scenarios to adapt to UAV detection tasks [18].

Although these methods provide useful designs for computational efficiency and long-range dependency modeling, the application of Mamba to RGB-T cross-modal small object detection remains underexplored. Therefore, this paper introduces a Wavelet Interactive Mamba module tailored for cross-modal feature denoising and interaction, targeting the specific demands of UAV small object detection scenarios.

### 2.2. Research Status of Multimodal Object Detection

Multimodal object detection improves robustness in complex environments by fusing multi-source perception data such as visible, infrared, and LiDAR. Existing methods mainly fall into CNN-based, Transformer-based, and recently Mamba-based hybrid frameworks.

Early CNN-based methods adopted either feature-level or decision-level fusion strategies. For instance, Tu et al. proposed ACENet to optimize RGB-T video detection performance through an Adaptive Context Enhancement Module [2]. For lightweight design, Jia et al. proposed LEFE-Net, which combines edge-aware depthwise separable convolution, edge feature enhancement, and an edge-integrated loss for salient object detection [3]. Wavelet-based representations have also been explored in RGB-T visual tasks to preserve texture details and exploit frequency-domain cues [19].

Transformer architectures are widely used in multimodal fusion because of their self-attention mechanisms. Li et al. introduced VoxFormer, a sparse voxel Transformer that uses camera images only for camera-based 3D semantic scene completion [20]. Minderer et al. developed the vision-language model OWL-ViT [21], which enables open-vocabulary detection through cross-modal pretraining. Despite their strong performance, Transformer-based models are limited by the quadratic complexity of self-attention, which increases computation when processing long sequences and complicates on-device deployment.

Mamba uses input-dependent selection to model long-range dependencies with linear complexity in sequence length. Its multimodal extensions include infrared–visible and camera-LiDAR fusion. Dong et al. proposed Fusion-Mamba to associate RGB-IR features in a hidden state space for cross-modal object detection [22]. Wang et al. proposed MambaFusion, which preserves height information during LiDAR voxel compression and uses a hybrid Mamba block for local and global camera-LiDAR feature fusion [23].

Although Mamba has shown promise in multimodal object detection, several issues remain open. In UAV RGB-T small object detection, targeted designs for thermal infrared noise interference and weak tiny-target features are still limited, which motivates this work.

Recent work makes the distinction between components and their coupling important. WaveNet uses wavelets and knowledge distillation for RGB-T salient object detection rather than selective state-space detection [19]. Fusion-Mamba models RGB-IR interaction in a hidden state space without frequency-band separation [22]. WaveMamba also combines DWT and Mamba for RGB-IR detection, but uses channel-swapping Mamba fusion for low-frequency bands, an absolute-maximum rule for high-frequency bands, and IDWT in the detection head [24]. In WIM-Net, interacted low-frequency features instead modulate high-frequency coefficients before reconstruction, and CMSF subsequently fuses the reconstructed features. EMFusionNet combines wavelets and Mamba for nighttime infrared–visible image fusion rather than detection [25].

For UAV tiny-object detection, AMSDet selects modality information before fusion [26], whereas the present architecture retains both inputs and attenuates frequency components. Tang et al. combine bidirectional fusion with a deformation-sensitive loss to improve tiny-object localization [27]. Their boundary objective is complementary to the present denoising design. Multiview infrared self-supervised representation learning addresses fine-grained ship recognition rather than paired RGB-T object localization [28]. IV-YOLO is a YOLOv8-based dual-branch RGB-IR detector that uses bidirectional pyramid feature fusion and Shuffle-SPP to combine complementary visible and infrared cues [29]. Its published configuration contains 4.31M parameters. IV-YOLO is additionally evaluated on RGBT-Tiny and DroneVehicle in Section 4.3.

## 3. Method

Combining RGB images with thermal information improves low-altitude remote sensing and object localization, but thermal noise, modal imbalance, and weak tiny-target features remain challenging. WIM-Net combines wavelet-based frequency filtering, state-space interaction, and complementary spatial fusion in a compact RGB-T detector.

### 3.1. Overall Network Architecture

**Figure 1:**
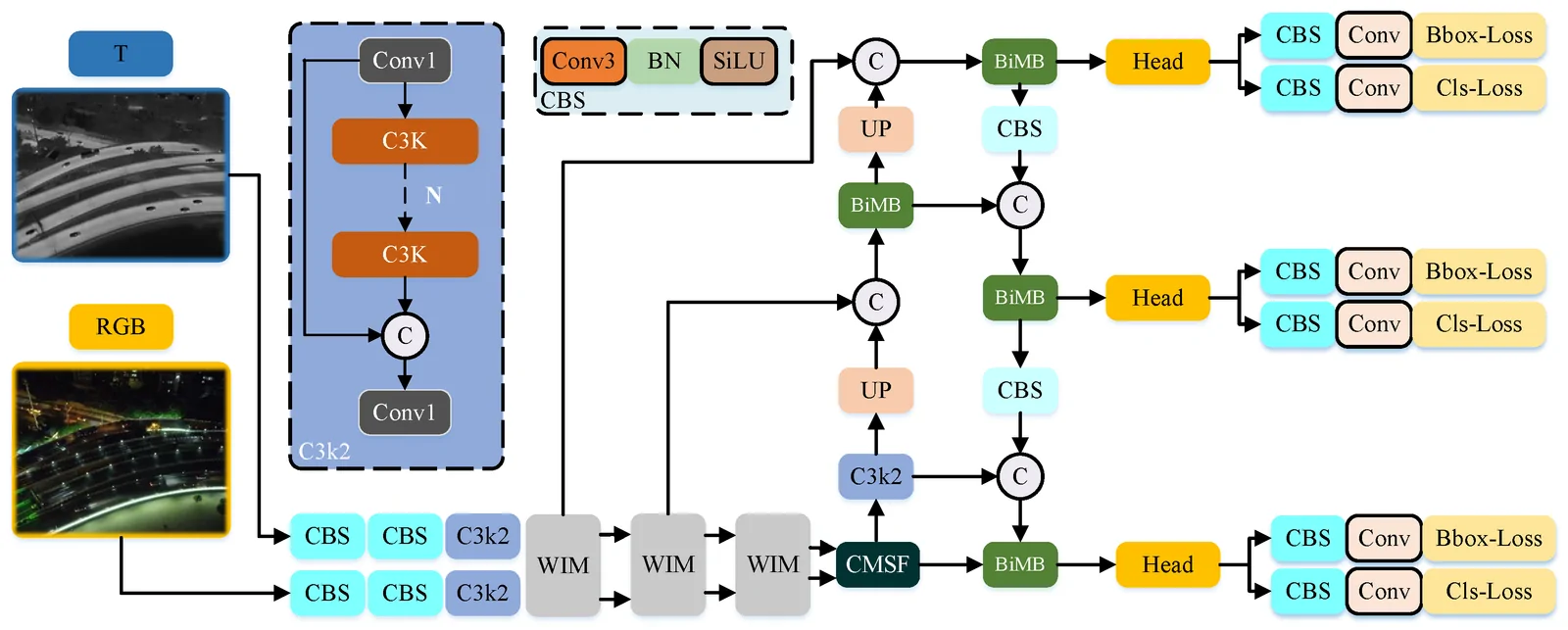
Overall architecture of the proposed WIM-Net. The network adopts a dual-branch input structure, performs denoising interaction and adaptive fusion through cascaded WIM modules and a CMSF module, and outputs detection results through the detection head.

The proposed WIM-Net adopts a dual-branch input structure as shown in Figure 1. One branch processes three-channel visible images, while the other branch processes single-channel thermal infrared images. Each branch first passes through two CBS convolution layers (composed of convolution, batch normalization, and the SiLU activation function) and a C3k2 module, which is a lightweight bottleneck structure with a convolution kernel size of 2, to complete initial feature extraction.

The features from both branches are then fed into three cascaded WIM modules for multi-scale frequency-domain denoising and cross-modal semantic interaction. Because the cascaded WIM modules operate at different feature scales, they can support targets of varying sizes. The interconnections between branches allow for feature exchange between modalities and strengthen tiny-target representations.

After three rounds of cross-modal interaction within the WIM modules, the features from both branches are fused by a CMSF module for adaptive complementary enhancement of heterogeneous features. The fused features are subsequently processed by another C3k2 module for further feature refinement. The network then diverges into three pathways, which are connected through a series of modules including a concatenation module, upsampling modules, and bidirectional Mamba modules, to construct a multi-scale feature pyramid. Each of these three pathways is connected to a detection head. Finally, the outputs are processed through convolution modules to compute the bounding box regression and classification losses, producing the detection results.

### 3.2. Wavelet Interactive Mamba Module

The WIM module is used for noise suppression and cross-modal semantic interaction. Unlike traditional single-modal wavelet denoising methods that process frequency components independently, this module couples discrete wavelet decomposition with cross-modal Mamba interaction and uses shared semantic information to guide high-frequency noise filtering while preserving target-related features.

**Figure 2:**
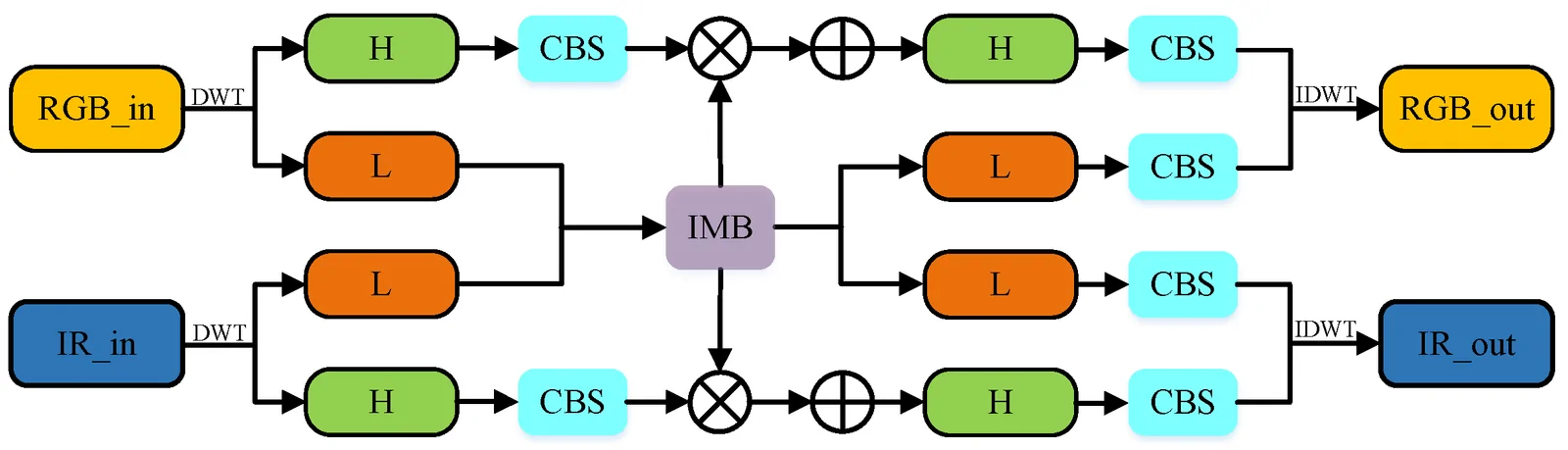
Structure of the WIM module.

The structure of the WIM module is shown in Figure 2. First, the features from the two input sources are fed into a feature decomposition layer based on a single-level discrete wavelet transform with the Haar wavelet, which is chosen because its shortest filters minimize hardware overhead and match the goal of suppressing noise while retaining edges. The input feature map of each modality is decomposed into one low-frequency sub-band and three high-frequency sub-bands through low-pass and high-pass filtering in horizontal and vertical directions, separating noise components from valid semantic information in the frequency domain. Specifically, the wavelet transform decomposition process can be formulated as follows:

(1) $$\left\{\begin{array}{c} F_{\mathrm{LL}}^{m}=F^{m}*\psi_{L}*\psi_{L}^{T}\\ F_{\mathrm{LH}}^{m}=F^{m}*\psi_{L}*\psi_{H}^{T}\\ F_{\mathrm{HL}}^{m}=F^{m}*\psi_{H}*\psi_{L}^{T}\\ F_{\mathrm{HH}}^{m}=F^{m}*\psi_{H}*\psi_{H}^{T} \end{array}\right.,$$

where m∈{RGB,T} denotes the modality index, $\psi_{L}$ and $\psi_{H}$ represent the low-pass and high-pass wavelet filter kernels respectively, * denotes the convolution operation, $F_{\mathrm{LL}}^{m}$ denotes the low-frequency sub-band containing main semantic information, and $F_{\mathrm{LH}}^{m}$, $F_{\mathrm{HL}}^{m}$, and $F_{\mathrm{HH}}^{m}$ denote three high-frequency sub-bands containing edge, texture and noise information respectively.

The decomposed feature components then enter a dual-branch processing pipeline. First, the low-frequency components of the two modalities are simultaneously fed into the Mamba Interaction (MI) module to model cross-modal global dependencies, and obtain the interactively enhanced low-frequency features of visible and thermal infrared modalities. This process can be formulated as follows:

(2) $$\left[F_{\mathrm{RGB},L}^{i},F_{T,L}^{i}\right]=\mathrm{MI}\left(F_{\mathrm{LL}}^{\mathrm{RGB}},F_{\mathrm{LL}}^{T}\right),$$

where $F_{\mathrm{RGB},L}^{i}$ and $F_{T,L}^{i}$ denote the interacted low-frequency features of visible and thermal infrared modalities respectively, and MI(·) denotes the Mamba interaction operation.

In the visible branch, the high-frequency components first undergo preprocessing by a CBS convolution module. The interacted low-frequency feature is then converted into a semantic soft gate by a 1×1 convolution, batch normalization and a Sigmoid function. This produces a bounded, data-dependent attenuation weight at each spatial location and channel. The Sigmoid output is not a calibrated probability of target structure. The preprocessed high-frequency components are multiplied element-wise with this semantic soft gate to obtain the noise-suppressed high-frequency visible components. This process can be formulated as follows:

(3) $$G^{\mathrm{RGB}}=\sigma \left(\mathrm{BN}\left(W_{g}*F_{\mathrm{RGB},L}^{i}\right)\right),$$

(4) $$F_{\mathrm{RGB},H}^{i}=\alpha \left(F_{\mathrm{RGB},H}\right)⊙G^{\mathrm{RGB}},$$

where α(·) denotes the CBS convolution layer composed of 3×3 convolution, batch normalization and the SiLU activation function, ⊙ denotes element-wise multiplication, σ(·) denotes the Sigmoid function, $G^{\mathrm{RGB}}\in \left[0,1\right]$ is the semantic soft gate, and $F_{\mathrm{RGB},H}$ denotes the original high-frequency components of the visible modality. Unlike a fixed scalar threshold, this operation applies a learned multiplicative soft mask derived from the interacted low-frequency features. It should be interpreted as attenuation rather than a formal wavelet shrinkage threshold. Guided by low-frequency semantic information, high-frequency noise components are suppressed, while edge and texture features are retained.

Subsequently, the calibrated high-frequency feature $F_{\mathrm{RGB},H}^{i}$ is added to the initial visible high-frequency component as a residual connection to avoid feature degradation. The result is processed again by a convolution layer, and then, together with the bimodal low-frequency component processed sequentially by the MI module and convolution layer, undergoes an inverse discrete wavelet transform to reconstruct the final output visible feature:

(5) $$F_{\mathrm{RGB},\mathrm{out}}=\mathrm{IDWT}\left(F_{\mathrm{RGB},L}^{i},\alpha \left(F_{\mathrm{RGB},H}^{i}+F_{\mathrm{RGB},H}\right)\right),$$

where IDWT(·) denotes the inverse discrete wavelet transform operation. The inverse wavelet transform reconstructs the refined frequency-domain components in the spatial domain, yielding the denoised and semantically enhanced visible feature $F_{\mathrm{RGB},\mathrm{out}}$.

For the thermal branch, a symmetric structure consistent with the visible branch is adopted, and the final thermal output feature $F_{T,\mathrm{out}}$ is obtained through the same frequency-domain interaction and reconstruction process.

### 3.3. Mamba Interaction Module

The MI module is designed for efficient cross-modal global feature modeling and learns compact multimodal representations. Unlike the native unidirectional Mamba structure designed for single-modal sequences, this module adopts a bidirectional selective scanning mechanism and constructs a shared hidden state space for bimodal features, enabling cross-modal interaction with linear computational complexity.

**Figure 3:**
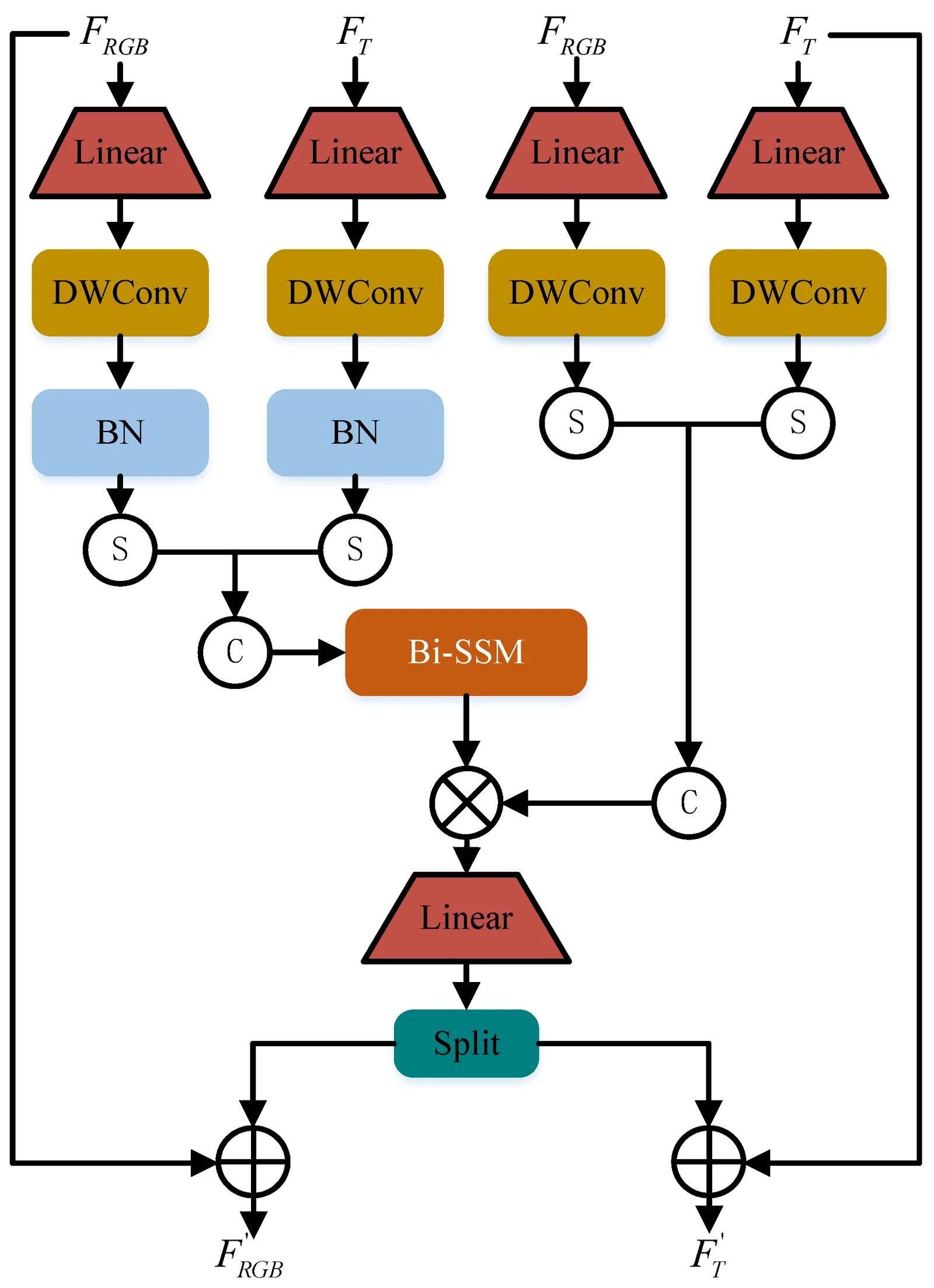
Structure of the Mamba Interaction module.

As illustrated in Figure 3, in the Mamba Interaction module, the dual-modal input features $F_{\mathrm{RGB}}$ and $F_{T}$ first go through two parallel processing branches. On the right branch, features first pass through a linear transformation layer, a depthwise convolution layer, a batch normalization layer, and the SiLU activation function, and are then aggregated and fed into the Bidirectional Selective Scan Model (BiSSM). The BiSSM extends the unidirectional state-space scanning of native Mamba to bidirectional sequence modeling, capturing context dependencies in both forward and reverse directions for visual feature modeling. The state-space update process of BiSSM can be formulated as follows:

A continuous state-space system can be written as $h^{\prime }\left(t\right)=Ah\left(t\right)+Bx\left(t\right)$ and y(t)=Ch(t)+Dx(t). In the selective discrete model, the step size $\Delta_{t}$ and input/output maps $B_{t},C_{t}$ depend on $x_{t}$, while *A* and the skip term *D* are learned time-independent parameters:

(6) $$\left\{\begin{array}{c} h_{t}={\bar{A}}_{t}h_{t-1}+{\bar{B}}_{t}x_{t}\\ y_{t}=C_{t}h_{t}+Dx_{t} \end{array}\right.,$$

where $h_{t}$ is the hidden state and $x_{t}$ is the input at step *t*. Under zero-order hold, ${\bar{A}}_{t}=\exp\left(\Delta_{t}A\right)$ and ${\bar{B}}_{t}=\left(\int_{0}^{\Delta_{t}}\exp\left(sA\right)\;ds\right)B_{t}$. The input-dependent quantities $\Delta_{t}$, $B_{t}$, and $C_{t}$ implement selection. Softplus constrains $\Delta_{t}$ to be positive. The learned *A* and *D* do not vary with *t*. A forward and a reverse scan use this recurrence before their outputs are combined.

On the left branch, the original bimodal features are processed by a linear layer and the SiLU activation function to retain local detail information. The output features of the right global branch and the left local branch are multiplied element-wise to obtain the intermediate aggregated feature:

(7) $$F_{\mathrm{mid}}^{M}=\beta \left(W_{l}\cdot \left[F_{\mathrm{RGB}},F_{T}\right]\right)⊙\Omega \left(D\left(W_{r}\cdot \left[F_{\mathrm{RGB}},F_{T}\right]\right)\right),$$

where $W_{l}$ and $W_{r}$ denote the weight matrices of the left and right linear layers respectively, β(·) denotes the SiLU activation function, D(·) denotes the depthwise convolution operation, and Ω(·) denotes the BiSSM operation. This design allows for adaptive information interaction between modalities and strengthens the consistency and distinctiveness of fused features.

After another linear projection layer, the features are split by the channel split operation to output the refined final features for each modality respectively:

(8) $$\left[F_{\mathrm{RGB}}^{\prime },F_{T}^{\prime }\right]=\mathrm{Split}\left(W_{o}\cdot F_{\mathrm{mid}}^{M}\right),$$

where $W_{o}$ denotes the weight matrix of the output linear projection layer, and Split(·) denotes the channel split operation.

With the aggregation mechanism and bidirectional selective scanning, the model performs cross-modal feature interaction with linear complexity, reducing computational overhead while improving multimodal feature discrimination.

### 3.4. Cross-Modal Synergistic Fusion Module

**Figure 4:**
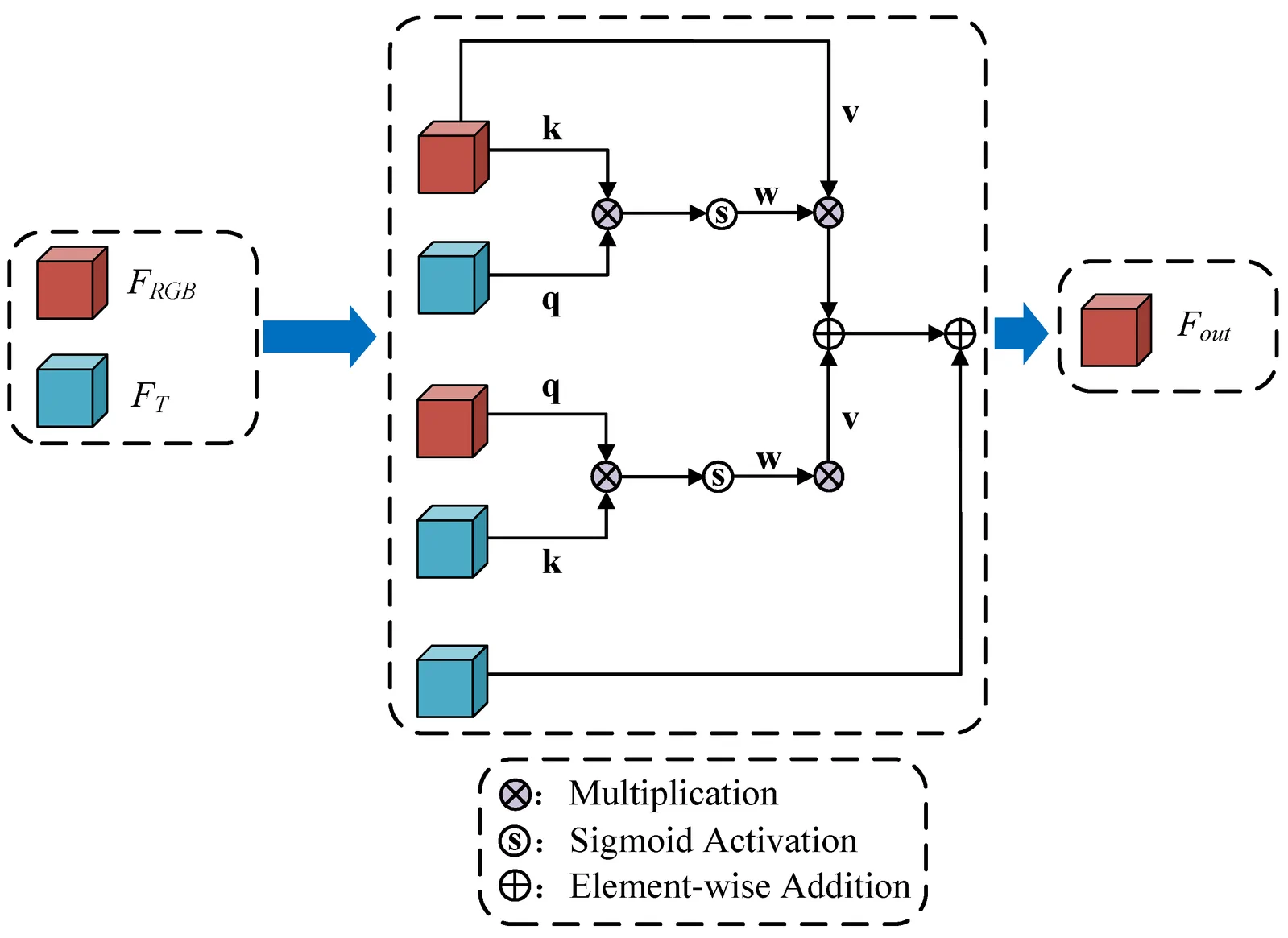
Structure of the CMSF module.

The structure of the CMSF module is shown in Figure 4. After multi-layer WIM processing, the bimodal features have been denoised and have undergone preliminary interaction. The CMSF module further enhances heterogeneous features through bidirectional mutual attention to improve weak tiny-target responses and reduce missed detections.

Mutual attention mechanisms can model bidirectional correlation between heterogeneous modalities and achieve adaptive feature enhancement. This module takes feature maps with the same spatial dimensions from visible and thermal images as inputs, and enhances bimodal features by modeling mutual-attention correlations between heterogeneous modalities.

Taking the visible feature $F_{\mathrm{RGB}}$ and thermal feature $F_{T}$ as examples, in the mutual attention operation, each modal feature is projected to the same channel dimension by modality-specific 1×1 convolutions and flattened over spatial positions to form the query and key tensors, i.e., $Q_{m}=W_{Q}^{m}*F_{m}$ and $K_{m}=W_{K}^{m}*F_{m}$, m∈{RGB,T}. No independent value tensor is introduced because the modal features weighted in Equation (10) directly play the role of values. Each modal feature then alternately acts as the query tensor and key tensor to conduct fine-grained correlation measurement between the modal features. The attention weight calculation process is as follows:

(9) $$\begin{array}{cc} M_{\mathrm{RGB}\to T} & =\sigma \left(Q_{T}⊙K_{\mathrm{RGB}}\right)\\ M_{T\to \mathrm{RGB}} & =\sigma \left(Q_{\mathrm{RGB}}⊙K_{T}\right) \end{array},$$

where ⊙ denotes element-wise multiplication and σ(·) is the Sigmoid activation. The maps $M_{\mathrm{RGB}\to T}$ and $M_{T\to \mathrm{RGB}}$ weight the opposite-modality features at corresponding positions and channels. Because the learned query and key projections may have signed outputs, a large product should be interpreted as learned compatibility rather than a calibrated probability or proof that both raw modalities have strong positive responses. For *N* spatial positions and *C* channels, the element-wise product and Sigmoid cost O(NC) after projection. Dense 1×1 query/key projections can cost $O(NC^{2})$. This remains linear in *N*, whereas full token-to-token dot-product attention forms an N×N matrix with $O(N^{2}C)$ work.

Subsequently, the two groups of mutual attention calculation results are fused, and the original thermal image feature is introduced as a residual tensor to be added to the fusion result. This design retains thermal features through the residual term and enhances them with complementary visible information. The final output of the CMSF module is calculated as follows:

(10) $$F_{\mathrm{out}}=\left(F_{\mathrm{RGB}}⊙M_{T\to \mathrm{RGB}}\right)⊕\left(F_{T}⊙M_{\mathrm{RGB}\to T}\right)⊕F_{T},$$

where ⊕ denotes the element-wise addition operation.

The purpose of the CMSF module is to model the correlation between visible and thermal features, emphasize shared discriminative cues, and suppress modality-specific irrelevant noise. In this way, the model focuses on cross-modal consistent information, improves the feature representation of the dual-modal fusion layer, and enhances the saliency of tiny targets.

## 4. Experiments

### 4.1. Datasets and Implementation Details

Experiments use two paired visible–thermal UAV datasets. RGBT-Tiny [1] contains approximately 93,000 frames across seven object categories and varied scenes, with most targets smaller than 16×16 pixels. DroneVehicle [30] contains 28,439 aligned image pairs and oriented annotations for five vehicle categories across different lighting conditions.

WIM-Net was trained on an NVIDIA RTX 3090 using Ubuntu 22.04, PyTorch 2.5.0, and CUDA 11.8. Training used SGD with momentum 0.937 and weight decay 0.0005 for 300 epochs, an initial learning rate of $1\times 10^{-4}$ with cosine decay, batch size 64, and 640×640 input images. The single-modal YOLOv11x baseline uses RGB images and the pretrained weights provided by the RGBT-Tiny authors, whereas WIM-Net uses a YOLOv11n-scale configuration. For DroneVehicle, the official training, validation, and test split is used. A 100-pixel border is removed before resizing, with box coordinates adjusted accordingly. Oriented ground-truth boxes are converted to horizontal boxes from their polygon extrema for evaluation.

### 4.2. Evaluation Metrics

Detection performance is assessed using Precision (P), Recall (R), F1-score, and mAP50. Precision measures the proportion of correctly detected positive samples among all detected positive samples, reflecting the false detection suppression ability of the model. Recall measures the proportion of correctly detected positive samples among all real positive samples, reflecting the missed detection suppression ability of the model. F1-score is the harmonic mean of Precision and Recall and measures their balance. mAP50 averages per-class Precision–Recall areas across confidence thresholds at IoU 0.5 and reflects overall detection performance. Together, these metrics evaluate both false detections and missed detections.

### 4.3. Comparative Experiment

Table 1 places the RGBT-Tiny and DroneVehicle comparisons side by side. The YOLOv11x baseline uses the RGBT-Tiny authors’ pretrained weights, while the proposed detector uses a YOLOv11n-scale training configuration. Their parameter counts therefore reflect different model scales as well as different modality designs. IV-YOLO is a lightweight YOLOv8-based RGB-IR detector with bidirectional pyramid feature fusion and Shuffle-SPP [29]. Its parameter count refers to the published configuration. All methods are evaluated using mAP50 at IoU 0.5, with DroneVehicle evaluated on its five vehicle categories. Bold values mark the best result in each dataset column.

Table 1 shows that WIM-Net leads in Precision and mAP50 on both datasets. On RGBT-Tiny, QFDet and CALNet retain higher Recall and F1-score. On DroneVehicle, QFDet has the highest Recall, and its rounded F1-score matches that of WIM-Net. IV-YOLO provides a compact dual-modal reference, although its parameter count refers to the published configuration. The different model scales prevent these comparisons from isolating architectural effects.

**Figure 5:**
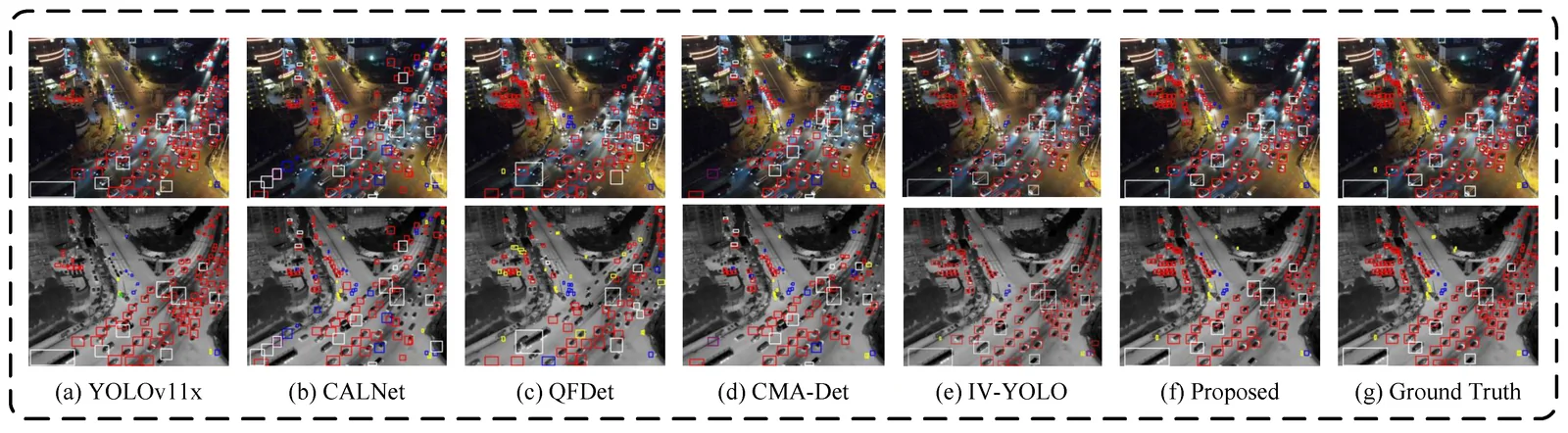
Visual comparison of detection results for different algorithms on night urban scenes. The upper and lower rows show visible and thermal images, respectively. Columns (**a**–**f**) show predictions and column (**g**) shows ground-truth annotations. Red, blue, yellow, white, and purple object bounding boxes denote cars, cyclists, pedestrians, buses, and ships, respectively.

**Figure 6:**
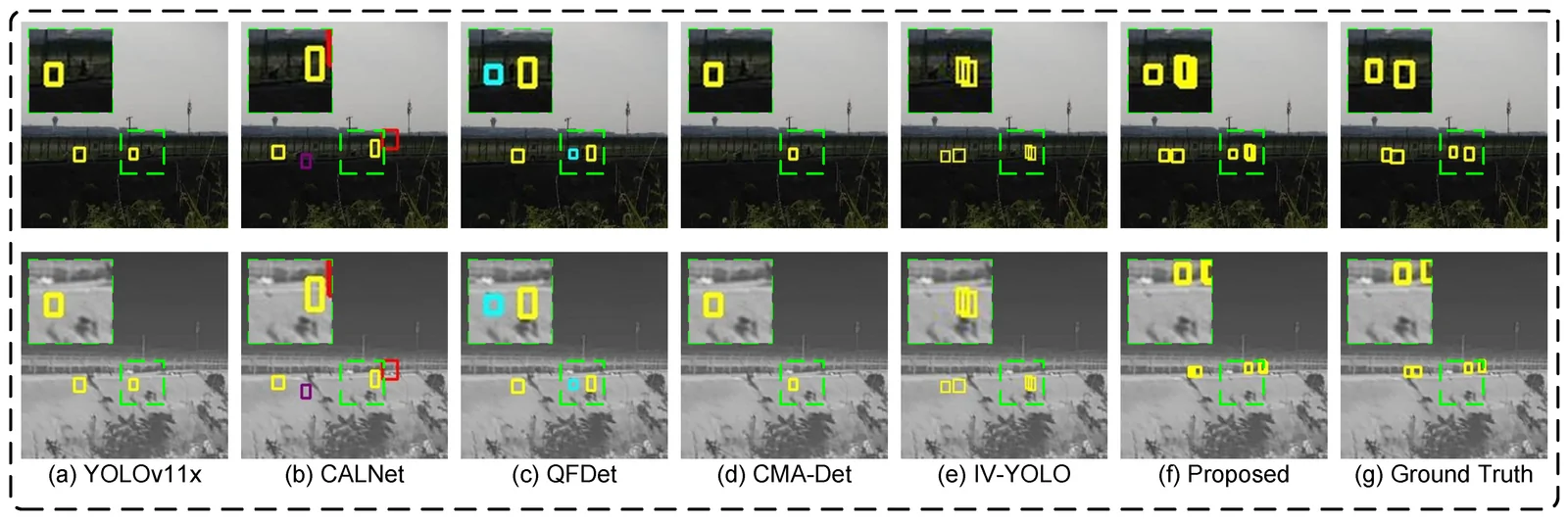
Visual comparison of extremely small object detection results. The upper and lower rows show visible and thermal images, respectively. Columns (**a**–**f**) show predictions and column (**g**) shows ground-truth annotations. Red, yellow, purple, and cyan object bounding boxes denote cars, pedestrians, ships, and planes, respectively. Green dashed rectangles mark the regions enlarged in the insets.

Figure 5 and Figure 6 illustrate dense road scenes and distant vehicles. YOLOv11x misses some small vehicles. Some dual-modal methods show background false detections or poorly fitted boxes. WIM-Net has fewer such visible errors in these examples, which do not establish a dataset-wide pattern.

### 4.4. Ablation Studies

Using the YOLOv11 dual-modal baseline as the base model, ablation experiments are conducted to evaluate the contribution of each module. The dual-modal baseline equips a YOLOv11 detector with two identical initial feature extractors, each comprising two CBS layers and one C3k2 module. One branch takes the three-channel RGB image, while the other takes the single-channel thermal image replicated to three channels at the stem. Their features are concatenated before the neck, and neither WIM nor CMSF is included. This dual-modal baseline differs from the single-modal YOLOv11 entry in Table 1. First, the WIM module is integrated into the baseline to perform frequency-domain decomposition of dual-modal features and separate valid high- and low-frequency features from noise information. The CMSF module is then introduced for adaptive complementary fusion of visible texture features and infrared thermal radiation features, forming the complete detection framework.

Table 2 compares the same four model configurations on both datasets. The DroneVehicle columns use its official five-category evaluation protocol.

On RGBT-Tiny, WIM alone improves Precision and mAP50 over the baseline, whereas CMSF alone improves Recall and mAP50 while slightly lowering Precision. The full model has the highest measured Precision and mAP50, but lower Recall than CMSF alone. This pattern is consistent with attenuation changing which weak and noisy responses survive fusion, but the aggregate metrics do not establish that mechanism. On DroneVehicle, the full model also has the highest mAP50, while CMSF alone has the highest Recall among the ablation variants. This reproduces the direction of the Precision–Recall trade-off across the two datasets.

**Figure 7:**
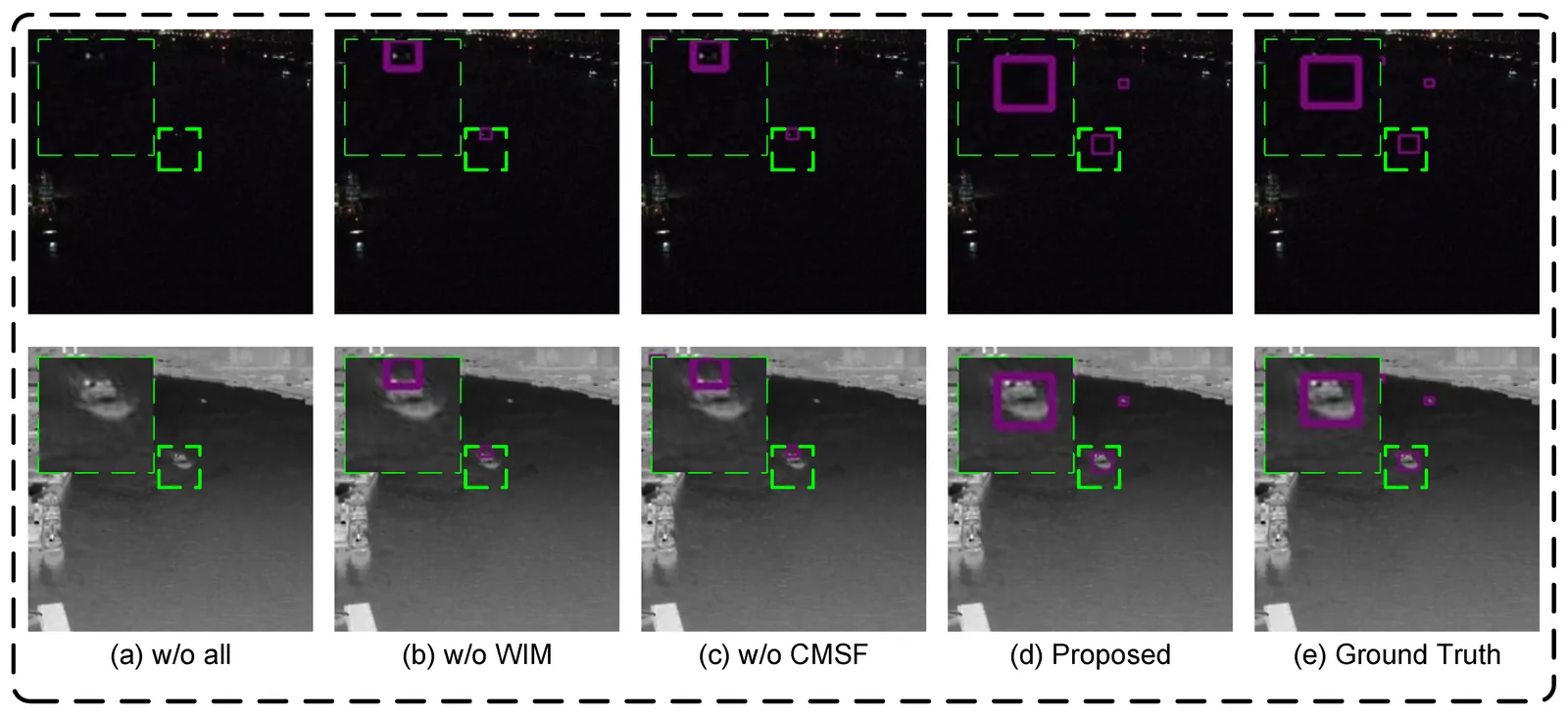
Visual comparison of scene ablation experiment results on river surface. The upper and lower rows show visible and thermal images, respectively. Purple object bounding boxes denote ships: predictions in columns (**a**–**d**) and ground-truth annotations in column (**e**). Green dashed rectangles mark the regions enlarged in the insets.

**Figure 8:**
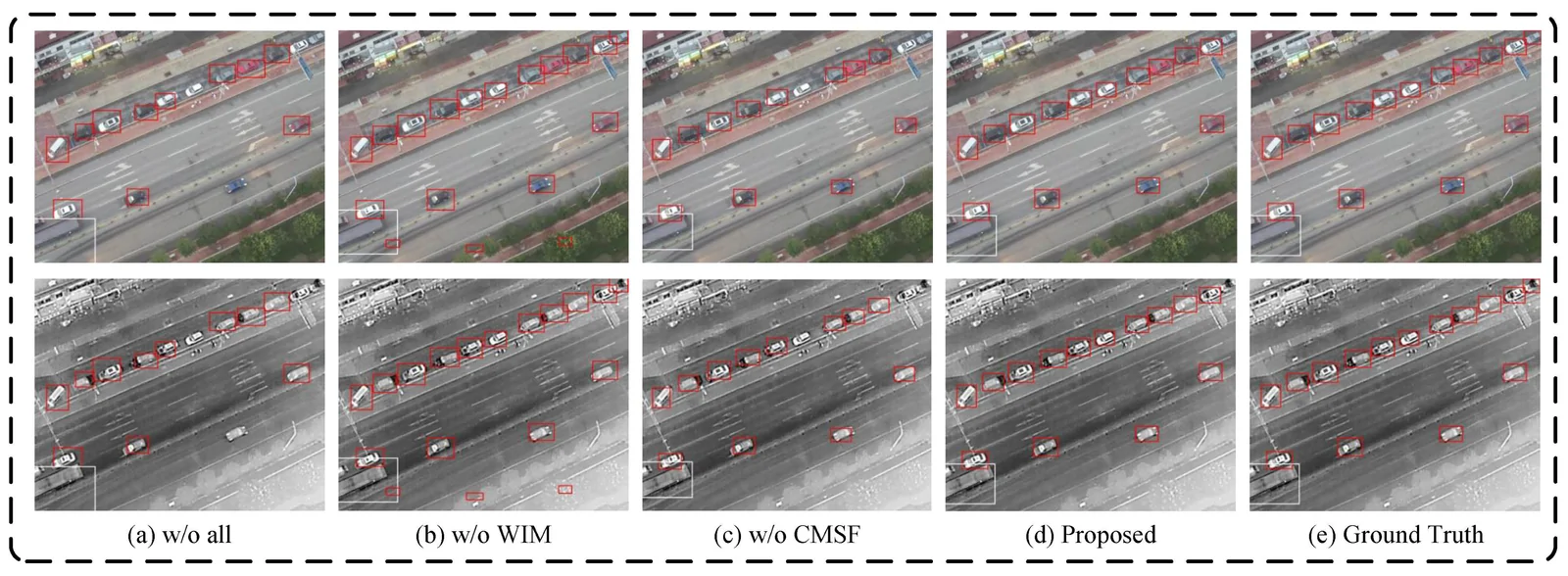
Visual comparison of ablation experiment results for extremely small object detection on road scenes. The upper and lower rows show visible and thermal images, respectively. Red and white object bounding boxes denote cars and buses, respectively: predictions in columns (**a**–**d**) and ground-truth annotations in column (**e**).

Figure 7 and Figure 8 illustrate examples in which WIM reduces visible false detections and CMSF helps recover some tiny objects. The RGBT-Tiny results in Table 3 qualify this impression: CMSF alone exceeds the full model on drone and ship but falls below the baseline on cyclist. The full model leads on bus, cyclist, and plane and is close to CMSF alone on car. These results measure category mAP50, not category Recall or boundary quality.

On DroneVehicle, the full model achieves the highest category-wise mAP50 for all five vehicle classes in Table 3. Both individual modules also improve each class over the baseline. Because this dataset contains only vehicles, it does not test pedestrian or cyclist generalization.

### 4.5. Deployment Efficiency

Table 4 reports model-only latency and full-process throughput for offline device tests using a batch size of 1 and 640×640 inputs. The embedded platform is an NVIDIA Jetson Orin NX running Linux and PyTorch. The wavelet transform and selective state-space operations are implemented in the same software stack. Model-only timing starts with a prepared tensor entering WIM-Net and includes DWT, IDWT, and selective scan. Full-process FPS additionally includes reading stored images, preprocessing, postprocessing, and writing annotated outputs. The Orin NX FP16 result is 30.8 ms per image and 25.1 full-process FPS, showing usable device throughput under this protocol. The model requires 32.6 GFLOPs per inference at this input size across all four configurations. Peak memory is reported in the table to characterize memory requirements. These offline tests do not measure an in-flight camera stream.

## 5. Discussion

Table 1 and Table 2 show a Precision–Recall trade-off. On RGBT-Tiny, WIM-Net’s Precision and mAP50 gains accompany lower Recall and F1-score than QFDet, CALNet, and the CMSF-only variant. On DroneVehicle, QFDet has higher Recall, while its rounded F1-score matches WIM-Net. Attenuation before fusion may suppress both noise and weak targets, but these aggregate metrics cannot establish which detections changed. Threshold and detection-level analyses are still needed. The cyclist and pedestrian results in Table 3 identify particularly difficult classes. In missed-detection-critical UAV tasks, the operating threshold should be selected for the required Recall rather than for peak Precision alone.

Boundary localization remains difficult for tiny non-rigid objects. Table 3 shows almost no gain for cyclists and only a modest gain for pedestrians. A shallow stem-to-WIM refinement path using parallel depthwise 3×3 and 5×5 convolutions could preserve detail, and a deformation-sensitive loss may improve box fitting [27]. Neither has been implemented or evaluated. The shallow path should be tested with a matched parameter budget and category-level localization metrics before any boundary-improvement claim is made. Failure of either sensor is also untested, and the thermal residual in CMSF does not guarantee graceful degradation. Blank, noisy, saturated, and spatially misregistered inputs should be evaluated separately, with reliability-aware gating considered if degradation is severe.

The RGBT-Tiny and DroneVehicle results in Table 1, Table 2 and Table 3 support evaluation across two datasets, and the Jetson Orin NX results in Table 4 characterize offline device throughput. DroneVehicle contains only vehicle classes, so pedestrian and cyclist generalization remains untested. The device results do not establish performance for a live airborne camera stream.

## 6. Conclusions

This paper presents WIM-Net, a compact RGB-T detector for low-altitude UAV imagery. WIM applies low-frequency-guided high-frequency attenuation before reconstruction, while CMSF fuses the reconstructed modal features. On RGBT-Tiny, the model obtains 52.0% mAP50 and 79.9% Precision with 10.4M parameters. Recall and F1-score remain below those of QFDet and CALNet. On DroneVehicle, the full model reaches 75.4% mAP50, and the offline Jetson Orin NX FP16 test reaches 25.1 full-process FPS. Live airborne runtime requires additional profiling.

Future work will measure energy use and live airborne latency, and test confidence calibration and shallow multi-scale refinement for stronger Recall and boundary localization. The proposed stem-level skip design and a deformation-sensitive loss require implementation and ablation before their effects can be claimed.

## Figures and Tables

**Table 1:** Comparative results on RGBT-Tiny and DroneVehicle. All metrics are percentages, with mAP50 at IoU 0.5. Parameter counts describe the specified model configurations. IV-YOLO’s 4.31M is from its published configuration.

Method	Params	RGBT-Tiny				DroneVehicle			
		P	R	F1	mAP50	P	R	F1	mAP50
YOLOv11x	61.5M	58.2	23.1	33.1	37.7	72.6	55.7	63.0	68.1
QFDet	60.3M	72.1	**34.6**	**46.8**	50.0	74.7	**63.5**	**68.6**	72.7
CALNet	153.6M	70.5	33.9	45.8	49.4	74.1	59.6	66.0	72.2
IV-YOLO	4.31M	74.8	25.0	37.5	48.6	75.8	60.4	67.2	74.0
CMA-Det	33.4M	61.2	26.7	37.2	40.9	71.9	57.8	64.1	69.5
Proposed	10.4M	**79.9**	25.2	38.3	**52.0**	**76.4**	62.2	**68.6**	**75.4**

*Note:* Bold numerical values indicate the highest value for each metric within each dataset; tied highest values are all shown in bold.

**Table 2:** Overall ablation results on RGBT-Tiny and DroneVehicle. P, R, F1 and mAP50 are percentages.

	RGBT-Tiny				DroneVehicle			
Variant	P	R	F1	mAP50	P	R	F1	mAP50
Baseline (w/o all)	75.0	23.2	35.4	49.0	73.4	59.2	65.5	72.3
CMSF only (w/o WIM)	74.5	**28.4**	**41.1**	51.1	72.8	**63.4**	67.8	74.1
WIM only (w/o CMSF)	76.7	24.5	37.1	50.6	75.2	60.5	67.1	73.5
Proposed (full)	**79.9**	25.2	38.3	**52.0**	**76.4**	62.2	**68.6**	**75.4**

*Note:* Bold numerical values indicate the highest value for each metric within each dataset; tied highest values are all shown in bold.

**Table 3:** Category-wise ablation mAP50 (%) on RGBT-Tiny and DroneVehicle. Base is the dual-modal baseline. CMSF and WIM indicate the single-module variants.

Category	Base	CMSF	WIM	Full
**RGBT-Tiny**				
car	56.6	60.1	58.8	60.0
drone	38.6	48.0	41.7	44.9
cyclist	32.3	26.2	29.4	32.4
pedestrian	40.2	40.1	42.8	42.4
bus	55.0	57.0	55.3	59.4
ship	59.8	66.1	64.2	62.8
plane	60.5	60.3	61.8	61.9
**DroneVehicle**				
car	78.0	79.5	79.1	80.5
truck	70.3	72.1	71.6	73.8
bus	73.1	74.6	74.2	76.0
van	72.4	73.9	73.3	75.1
freight car	67.9	70.2	69.5	71.6

**Table 4:** Offline device efficiency of WIM-Net at 640×640 input and batch size 1.

Metric	RTX 3090 FP32	Orin NX FP32	Orin NX FP16	Orin NX INT8
Model time (ms/img)	11.4	54.0	30.8	24.6
Full-process FPS	64.5	14.2	25.1	31.8
Peak memory (MB)	2347	2261	1623	1368

## Data Availability

The code and trained models generated in this study are available from the corresponding author upon reasonable request.

## Abbreviations

The following abbreviations are used in this manuscript:

WIM-Net	Wavelet Interactive Mamba Fusion Network
WIM	Wavelet Interactive Mamba
CMSF	Cross-Modal Synergistic Fusion
MI	Mamba Interaction
BiSSM	Bidirectional Selective Scan Model
UAV	Unmanned Aerial Vehicle
RGB-T	Red–green–blue and thermal imaging
mAP	Mean Average Precision
CNN	Convolutional Neural Network
SSM	State-Space Model
DWT	Discrete Wavelet Transform
IDWT	Inverse Discrete Wavelet Transform
CBS	Convolution-BatchNorm-SiLU
RPN	Region Proposal Network
FLOPs	Floating-Point Operations
